# NAD(P) transhydrogenase isoform distribution provides insight into apicomplexan evolution

**DOI:** 10.3389/fevo.2023.1216385

**Published:** 2023-06-28

**Authors:** Annie Z. Tremp, Sadia Saeed, Johannes T. Dessens

**Affiliations:** 1 Department of Infection Biology, Faculty of Infectious and Tropical Diseases, London School of Hygiene & Tropical Medicine, Keppel Street, London WC1E 7HT, United Kingdom

**Keywords:** Transhydrogenase, isoform, TSAR, alveolates, Apicomplexa, Plasmodium

## Abstract

Membrane-located NAD(P) transhydrogenase (NTH) catalyses reversible hydride ion transfer between NAD(H) and NADP(H), simultaneously translocating a proton across the membrane. The enzyme is structurally conserved across prokaryotes and eukaryotes. In heterotrophic bacteria NTH proteins reside in the cytoplasmic membrane, whereas in animals they localise in the mitochondrial inner membrane. Eukaryotic NTH proteins exists in two distinct configurations (isoforms) and have non-mitochondrial functions in unicellular eukaryotes like *Plasmodium*, the causative agent of malaria. In this study, we carried out a systematic analysis of *nth* genes across eukaryotic life to determine its prevalence and distribution of isoforms. The results reveal that NTH is found across all major lineages, but that some organisms, notably plants, lack *nth* genes altogether. Isoform distribution and phylogenetic analysis reveals different *nth* gene loss scenarios in apicomplexan lineages, which sheds new light on the evolution of the Piroplasmida and *Eimeriidae*.

## 1 Introduction

**Figure 1:**
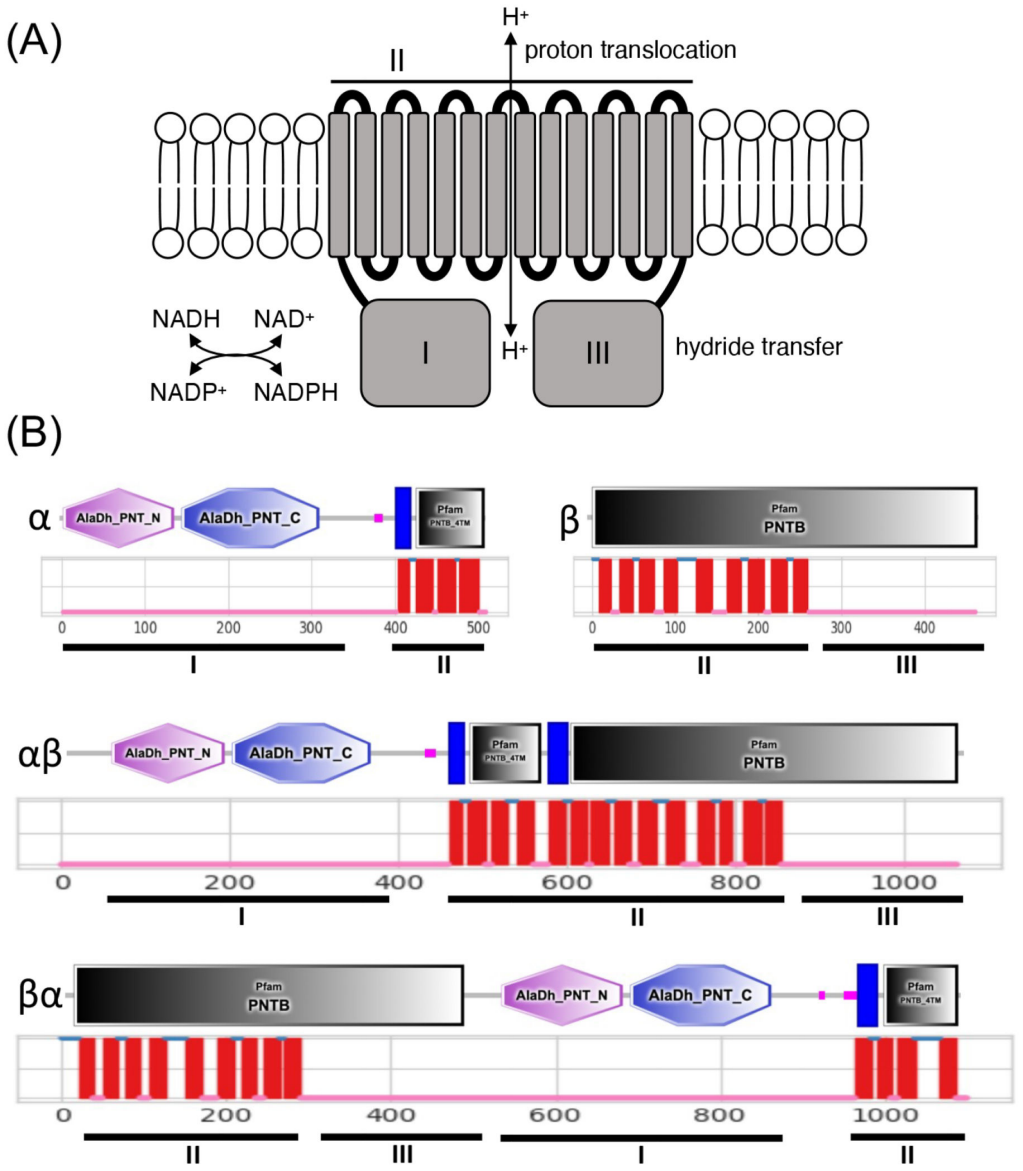
Structure of membrane bound NTH proteins. (A) Predicted functions of NTH in the lipid bilayer showing functional domains I-III and corresponding functional activities. (B) Organisation of functional NTH domains I-III in different NTH isoforms. Depicted are SMART and Pfam domains (https://smart.embl-heidelberg.de) (top) and predicted transmembrane helices (https://dtu.biolib.com/DeepTMHMM) (middle).

Proton-translocating NAD(P) transhydrogenase (NTH), also known as nicotinamide nucleotide transhydrogenase (NNT) or pyridine nucleotide transhydrogenase (Pnt), is an integral membrane protein that catalyses the reversible hydride ion transfer between NAD(H) and NADP(H), whilst simultaneously translocating a proton across the membrane in which it is embedded (Fig. 1A) (Anderson and Fisher, 1981; Hou et al., 1990; Hatefi and Yamaguchi, 1996). The enzyme is found in both prokaryotes and eukaryotes and is structurally conserved, possessing three functional domains: domain I that binds NAD(H); domain II that is made up of the transmembrane (TM) helices and has proton translocating activity; and domain III that binds NADP(H) (Fig. 1A). In animals, NTH proteins reside in the mitochondrial inner membrane, whereas in heterotrophic bacteria they localise in the cytoplasmic membrane (Hatefi and Yamaguchi, 1996; Jackson, 2003), possibly reflecting the evolutionary origin of the mitochondrion from a proteobacterial endosymbiont (Andersson et al., 1998; Gray et al., 1999). The physiological role of NTH is widely considered to be generation of NADPH (the reduced form of NADP), an important cofactor used for redox homeostasis and for NADPH-dependent enzymatic activity.

Bacterial NTH (PntAB) is a composite enzyme composed of an α subunit (PntA) and β subunit (PntB) (Fig. 1B) encoded by *pntA* and *pntB* genes, respectively, typically located within polycistronic operons. In some prokaryotes, the α subunit is further split into α1 and α2 subunits (Leung et al., 2015). Domain I is located on the α subunit; domain II corresponds to the combined TM helices of subunits α and β; and domain III is located on the remainder of subunit β (Fig. 1B). In contrast to bacterial NTH, eukaryotic NTH is formed by a single protein chain corresponding to a fusion of the α subunit portion at its carboxy-terminus with the amino-terminus of the β subunit portion (αβ configuration) (Fig. 1B). We recently showed that *Plasmodium* species encode a single NTH protein that possesses an inverted structure in which the β subunit portion sits amino-terminally to the α subunit portion (βα configuration) (Saeed et al., 2020) (Fig. 1B). The same was found for NTH of the unicellular eukaryote *Entamoeba histolytica* (Weston et al., 2002).

We have shown that *Plasmodium* NTH does not reside in the mitochondrion, but is instead found localised in crystalloid and apicoplast organelles (Saeed et al., 2020). These findings demonstrate that the NTH enzyme is in fact not functionally confined to mitochondria and has a wider and possibly more diverse role in eukaryotic biology. For this study, we conducted a systematic analysis of *nth* genes across eukaryotic life to determine the prevalence of proton-translocating NTH and the distribution of its isoforms. The distinct underlying protein configurations of eukaryotic NTH (Fig. 1) provide an additional tool for evolutionary biology as it can be combined with conventional phylogenetic data. We use this approach to consider the evolutionary trajectory of the Apicomplexa.

## 2 Materials & Methods

NTH amino acid sequences were identified using the *Plasmodium falciparum* NTH protein sequence as query in BlastP searches of nonredundant protein databases (including GenPept, RefSeq, Swiss-Prot, PIR, PRF, and PDB), or TblastN searches of transcriptome shotgun assembly (TSA) or whole genome shotgun (WGS) databases at NCBI (https://ncbi.nlm.nih.gov) and VEuPathDB (https://veupathdb.org) web servers. Sequences were quality assessed by SMART/Pfam domain analysis (Letunic et al., 2021) (https://smart.embl-heidelberg.de) and DeepTMHMM transmembrane topology prediction (Hallgren et al., 2022) (https://dtu.biolib.com/DeepTMHMM) to conform with proton-translocating NTH characteristics (Fig. 1B). Geneious Prime software was used to carry out βα-type NTH phylogenetic reconstructions using default settings. Specifically, amino acid multiple sequence alignment was conducted with Clustal Omega (Sievers et al., 2011) (full distance matrix for guide-tree calculations and cluster size 100, mBed algorithm); PhyML (Guindon et al., 2010) was used for maximum likelihood tree building (Le Gascuel substitution model); MrBayes (Huelsenbeck and Ronquist, 2001) was used for Bayesian inference tree building (rate matrix: Poisson; rate variation: gamma, 4 categories; chain length: 1,100,000; heated chains: 4; Heated chain temp: 0.2; subsampling frequency: 200; burn-in length: 100,000; unconstrained branch lengths). *Vitrella brassicaformis* NTH (Vbra_8937) was used as outgroup for apicomplexan phylogenies, being a close relative (Chromerida). *Stygiella incarcerata* NTH (ANM86865) was used as outgroup for TSAR phylogenies, as a βα-type NTH from a non-TSAR clade of unicellular eukaryotes (Discoba).

## 3 Results

### 3.1 Distribution of NTH across eukaryotes

It is important to note that apparent absence of *nth* genes from taxa could be the result of incomplete genome/transcriptome coverage. Absence is therefore putative unless specified otherwise.

#### Amorphea

This taxon is composed of opisthokonts (animals, fungi and their unicellular relatives) and Amoebozoa (classification according to (Adl et al., 2019; Burki et al., 2020)). We found *nth* genes exclusively of the αβ configuration in all animals (Metazoa) as diverse as stony corals to warm-blooded vertebrates (Table 1, Table S1). The fungi also encode NTH exclusively of the αβ configuration (Table 1, Table S1), but in contrast to animals not all fungal taxa encode NTH, including yeasts. In the Amoebozoa, a group of amoeboid protists sister to the opisthokonts, we found taxa encoding either αβ-type or βα-type NTH (Table 1, Table S1).

#### Archaeplastida

This taxon includes plants (Streptophyta) and green, red and glaucophyte algae, which all contain photosynthetic plastids (chloroplasts) derived from primary endosymbiosis with a cyanobacterial ancestor (Raven and Allen, 2003). In sharp contrast to animals, we found no NTH proteins in plants (Table 1). Nonetheless, unsegmented *nth* genes of either a αβ or βα configuration are found in some primary algae belonging to Rhodophyta and Chlorophyta (Table 1, Table S1).

#### TSAR

The constituent lineages of TSAR: Telonemia, Stramenopila, Alveolata and Rhizaria collectively are estimated to encompass up to half of all eukaryotic species. We found a mixed picture in this group of unicellular eukaryotes, with some genera lacking NTH, some having NTH of αβ configuration, and others possessing *nth* genes encoding βα-type NTH (Table 1, Table S1). Few genera possess genes encoding both αβ-type and βα-type NTH.

#### Haptista

In this taxon of haptophyte algae we found organisms apparently without *nth* genes, as well as those encoding αβ-type NTH.

#### Cryptista

This taxon of unicellular eukaryotes includes organisms that encode αβ-type NTH (Table 1, Table S1).

#### Excavates

This is a newly proposed taxon that includes lineages Discoba, Metamonada and Malawimonadida (Burki et al., 2020). We identified several organisms in this clade encoding either αβ-type NTH or βα-type NTH (Table 1, Table S1).

#### CRuMs

This represents a newly proposed small clade combining several lineages of unicellular eukaryote: Collodictyonidae, Rigifilida and *Mantamonas*. We found no *nth*-encoding genes in organisms belonging to this clade (Table 1, Table S1).

### 3.2 Distribution of NTH among Apicomplexa

The Apicomplexa constitute a large eukaryotic lineage made up of microbial endosymbionts of animals, that includes medically important parasites such as *Plasmodium* and *Toxoplasma* species (causative agents of malaria and toxoplasmosis, respectively), as well as important pathogens of livestock such as *Eimeria, Theileria* and *Babesia* species. Though they constitute a diverse group of organisms with thousands of species across hundreds of genera, they share distinctive secretory organelles and cytoskeletal structures enabling motility, host cell interaction, invasion and egress (Frenal et al., 2017). Apicomplexans also share, with few exceptions, a relic four-membrane plastid called the apicoplast, which is of algal origin but no longer has photosynthetic activity (McFadden et al., 1996; Janouskovec et al., 2010; Lim and McFadden, 2010). Apicomplexans can be placed into different categories:

#### (1) ‘Core’ Apicomplexa

Organisms in this category are known for causing important human and animal diseases and are therefore best studied. They include two major lineages:

##### Hematozoa

These are arthropod-transmitted intracellular blood parasites of vertebrates. Insect-transmitted Haemosporida (syn. Heamospororida) possess a single NTH protein of the βα-type that is both orthologous and syntenic between the three haemosporidan genera investigated (Table S2). By contrast, we did not find any NTH-encoding genes in organisms belonging to the tick-transmitted Piroplasmida (syn. Piroplasmorida) (Table S2). The robust sequence coverage of *Babesia* and *Theileria* genomes indicates that this reflects a genuine absence of NTH proteins in piroplasmids.

##### Coccidia

The coccidians constitute non-vector-transmitted intracellular gut parasites of vertebrates and are grouped into families *Sarcocystidae* (e.g. genera *Toxoplasma, Sarcocystis, Neospora, Hammondia, Besnoitia, Cystoisospora*) and *Eimeriidae* (e.g. genera *Eimeria* and *Cyclospora*). This grouping is supported by the distinct representation of *nth* genes in these organisms, with the *Sarcocystidae* encoding two distinct βα-type proteins, while the *Eimeriidae* encode only a single NTH of the βα conformation (Table S2). The latter copy is in fact both orthologous and syntenic with one of the *nth* genes of the *Sarcocystidae*, confirming the close evolutionary relationship between these two coccidian lineages.

#### (2) ‘Basal’ Apicomplexa

Organisms in this category constitute deep-rooted lineages, including:

##### Cryptosporidia

This lineage contains organisms of the genus *Cryptosporidium* that were once regarded coccidians based on their similar biology, but were later removed from the coccidian lineage based on molecular phylogenetic evidence. *Cryptosporidium* species possess two distinct and conserved *nth* genes, both of the βα configuration (Table S2).

##### Gregarinia

Species belonging to this apicomplexan clade constitute a diverse group of extracellular, non-parasitic endosymbionts found in a wide range of terrestrial, marine and freshwater invertebrates (Rueckert et al., 2019). We identified both αβ- and βα-type NTH proteins in this lineage (Table S2).

##### Marosporida

The Marosporida form a newly proposed deep-rooted apicomplexan monophyletic lineage (Mathur et al., 2021). Its taxa include species of the genera *Rhytidocystis, Margolisiella, Aggregata* and *Merocystis*, intracellular endosymbionts found in a variety of marine invertebrates including molluscs, annelids, whelks and crustaceans (Miroliubova et al., 2020). Analysis of transcriptomic sequence from *Rhytidocystis* species isolated from *Ophelia limacina* and *Travisia forbesii* (Janouskovec et al., 2019) identifies at least four distinct *nth* genes encoding both αβ- and βα-type NTH proteins (Table S2).

#### (3) ‘Other’ Apicomplexa

This category of apicomplexans includes the Nephromycida, species which are found in marine ascidian tunicates with which they have suspected mutualistic (e.g. genus *Nephromyces*) or parasitic (e.g. genus *Cardiosporidium*) relationships. Species of Nephromycida are unusual in that they accommodate and rely on bacterial endosymbionts (Hunter et al., 2020; Paight et al., 2022). At least two distinct βα-type NTH-encoding genes are present in the assembled genome of *Cardiosporidium cionae* species isolated from the hemolymph of the tunicate *Ciona intestinalis* (Hunter et al., 2020) (Table S2). In addition, we identified at least three distinct βα-type NTH-encoding genes from the metagenome of *Nephromyces* species isolated from the renal sac of the tunicate *Molgula occidentalis* (Munoz-Gomez et al., 2019) (Table S2).

### 3.3 NTH phylogeny

**Fig. 2:**
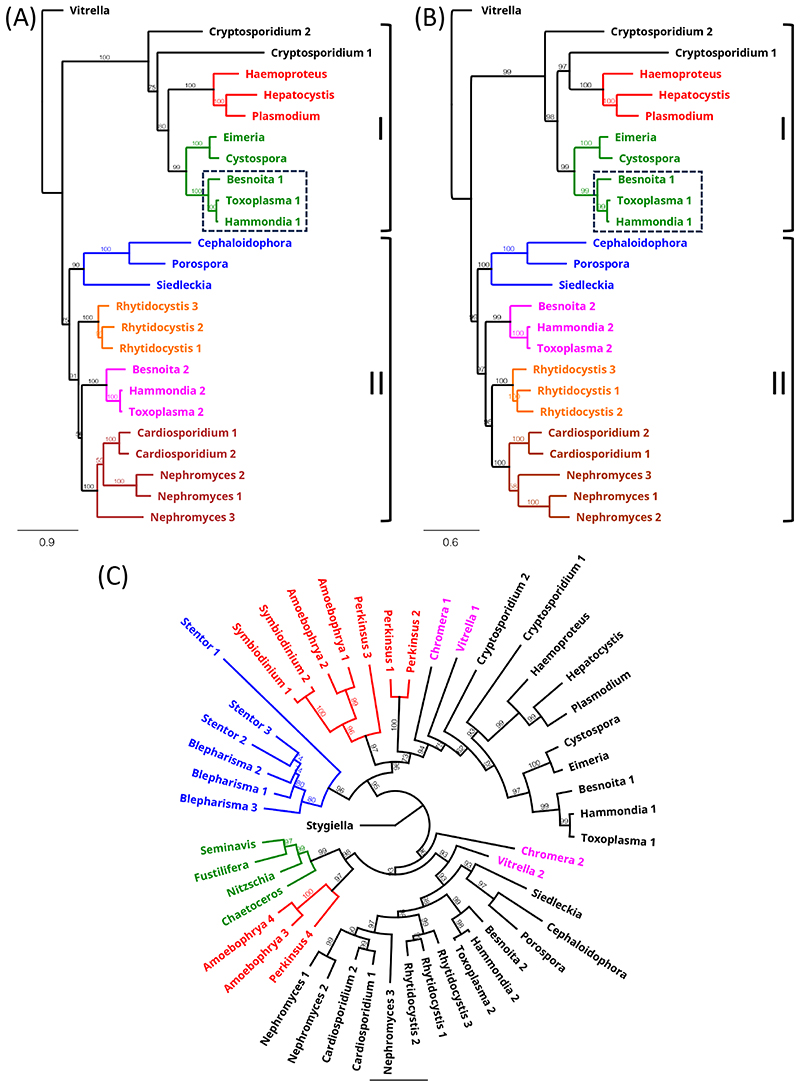
Phylogeny of βα-type NTH proteins. Phylogenetic reconstruction of apicomplexan NTH sequences using maximum likelihood (A) and Bayesian inference (B) methods. Colours in (A) and (B) represent Haemosporida (red), Coccidia (green, *Sarcoscystidae* indicated with dashed box), Gregarinia (blue), Marosporida (orange), Nephromycida (maroon) and *Sarcocystidae* (pink). Outgroup: *Vitrella* (Chromerida). Numbers behind genus names indicate distinct NTH homologues found. Paralogous groups I and II are indicated on right hand side. (C) Circular phylogram generated by Bayesian inference of apicomplexan NTH sequences (black) with inclusion of sequences from chromerid (pink), stramenopile (green), ciliate (blue) and dinoflagellate/perkinsid (red) lineages. Outgroup: *Stygiella* (Discoba). Bootstrap support (n=100) (A) and consensus support percentages (B, C) are shown to indicate branch support.

To shine more light on the underlying evolutionary relationships of the Apicomplexa, we conducted a phylogenetic examination of their βα-type NTH sequences, which are shared between all apicomplexan lineages except piroplasmids (Table S2). Phylogenetic reconstruction using maximum likelihood or Bayesian methods produced very similar trees, clustering the NTH sequences according to established apicomplexan groupings (Fig. 2A, B). The only exception to this were the two cryptosporidian NTH sequences, indicating that the event that gave rise to these two NTH copies occurred early in apicomplexan evolution. Notably, βα-type NTH sequences split into two groups, I and II (Fig. 2A, B), pointing to their descendance from ancestral *nth* paralogues. The *Sarcocystidae* are the only apicomplexan lineage to have retained *nth* copies from both paralogous groups (Fig. 2A, B). By contrast, the Cryptosporidia, Haemosporida and *Eimeriidae* lineages have lost the group II *nth* copy, whereas the Gregarinia, Marosporida and Nephromycida lineages have lost the group I *nth* copy (Fig. 2A, B).

The closest living relatives of Apicomplexa are the Chromerida (now grouped within Colpodellida lineage): coral-associated photosynthetic algae whose plastids share many similarities with the apicoplast including four limiting membranes (Moore et al., 2008; Obornik et al., 2012; Janouskovec et al., 2015). Given these features and the notion that the ancestral apicomplexan was itself capable of photosynthesis, the chromerids are considered to share a most recent common ancestor with the Apicomplexa, a view that is robustly supported by phylogenomics (Janouskovec et al., 2019; Mathur et al., 2019; Munoz-Gomez et al., 2019; Salomaki et al., 2021). This is indeed consistent with the NTH isoform distribution among chromerids, encoding both αβ-type and βα-type NTH (Table S1, Table S3). Inclusion of βα-type NTH sequences from the chromerid lineages *(Chromera* and *Vitrella*) in the phylogenetic reconstruction revealed similar paralogous origins shared with the Apicomplexa (Fig. 2C), indicating that the most recent common ancestor of apicomplexans and chromerids possessed at least two βα-type NTH paralogues (Table 2). Based on this NTH isoform distribution, different lineage-specific scenarios of *nth* gene loss/retention in extant apicomplexans can be inferred (Table 2). In addition to chromerids, other βα-type NTH sequences from the TSAR clade (Table S3) clustered broadly according to lineage and positioned basal to the Apicomplexa (Fig. 2C) in agreement with established phylogenies (Janouskovec et al., 2019; Mathur et al., 2019; Salomaki et al., 2021). NTH sequences from dinoflagellates and the closely related Perkinsidae (Zhang et al., 2011) were also found distributed across both paralogous βα-type NTH groups.

## 4 Discussion

Our systematic analysis of *nth* genes across eukaryotic life reveals that NTH is found across all major lineages, but equally that many organisms, notably plants, lack *nth* genes, indicating that the selective pressures on maintaining NTH activity vary widely between different organisms. Our analysis provides evidence for recent loss and recent gain of NTH-encoding genes. One example of *nth* gene loss is provided by the *Eimeriidae* lineage, which possesses only one *nth* gene that is orthologous and syntenic with an *nth* gene in the closely related *Sarcocystidae*. The second NTH copy present in the *Sarcocystidae*, which only shares distant homology with the first (Fig. 2A), is absent from the *Eimeriidae* (Table S2), providing evidence that the *Eimeriidae* evolved from the *Sarcocystidae* lineage within the coccidian clade. Another example of recent *nth* gene loss comes from the Piroplasmida (Table 2) that are lacking NTH altogether, whereas the closely related Haemosporida lineage possess NTH encoded by a single gene (Table 2). This observation strongly indicates that the Piroplasmida evolved from the haemosporidan lineage within the Hematozoa, and not the other way around. An example of recent gain of NTH-encoding genes via gene duplication comes from *Porospora* sp. that possess two virtually identical βα-type *nth* genes that are tandemly located, as well as three tandem and virtually identical αβ-type *nth* genes (Table 2, Table S2). We found no evidence to suggest that αβ-type *nth* genes readily convert into βα-type genes or *vice versa*, for example via rearrangement after gene duplication. Only older (unicellular) lineages possess αβ and βα-type *nth* genes, and among those very few organisms possess both αβ and βα-type NTH simultaneously (Table 1, Table S1). This suggests that events that led to the acquisition of the αβ- and βα-type *nth* genes happened early in eukaryotic evolution and that these isoforms have subsequently been maintained through vertical gene transfer/inheritance.

How did the unsegmented *nth* gene end up in eukaryotes? One possibility is that the eukaryotic unsegmented *nth* gene was obtained by lateral gene transfer from prokaryotic endosymbionts that gave rise to the mitochondrion (Andersson et al., 1998; Gray et al., 1999), followed by gene fusion. Increasing prevalence of gene fusions in eukaryotes over prokaryotes has also been observed for genes involved in purine biosynthesis (Chua and Fraser, 2020). There is evidence that mitochondrial evolution involved multiple bacterial endosymbionts (Baughn and Malamy, 2002) so lateral gene transfer could have occurred more than once. This scenario would offer an explanation for the apparent conservation of both location and function between prokaryotic and mitochondrial NTH, as well as for the creation of the two different NTH configurations.

Recent phylogenomic studies strongly support the grouping of coccidians, hematozoans, cryptosporidians and gregarines as four distinct monophyletic apicomplexan lineages (Janouskovec et al., 2019; Mathur et al., 2019; Salomaki et al., 2021). However, the evolutionary relationship between these four main apicomplexan clades remains controversial, Mathur et al supporting monophyly of Cryptosporidia with Coccidia and Hematozoa (Mathur et al., 2019); Janouskovec et al favouring monophyly of Gregarinia with Cryptosporidia (Janouskovec et al., 2019); and Salomaki and colleagues recovering monophyly of Gregarinia with Coccidia and Hematozoa (Salomaki et al., 2021). Among the Apicomplexa, only the gregarines and Marosporida possess NTH proteins of the αβ configuration, a feature shared with the chromerids (Table 2). It is tempting to speculate that a single event of αβ-type *nth* gene loss from the apicomplexan lineage, after the divergence of Marosporida and Gregarinia, led to the absence of αβ-type NTH from the other apicomplexan lineages (Table 2). This scenario would place the Marosporida and Gregarinia most basal in the apicomplexan phylogeny and thus favour the tree topology proposed by Mathur and colleagues (Mathur et al., 2019). However, it is also possible that αβ-type *nth* gene loss occurred multiple times during apicomplexan evolution, as appears to have been the case for the βα-type *nth* genes (Table 2), and therefore the question which apicomplexan lineage was first to diverge remains unresolved. Nonetheless, the NTH isoform distribution identified here (Table 2) argues against the proposed groupings of Nephromycida with Haemosporida and Piroplasmida, or of Cryptosporidia with the gregarines (Adl et al., 2019).

In phototrophic cyanobacteria, NTH is located in the thylakoid membranes (Kamarainen et al., 2017). However, the absence of NTH from all multicellular plants and most unicellular algae (Table 1, Table S1) indicates that NTH was not maintained in the cyanobacterium-derived chloroplasts. This contrasts with the apparent reliance on NTH in animals (Table 1). What could be the reason for this? One explanation is that plants, which are fully autotrophic, use a portion of the ATP generated during photosynthesis to reduce NADP^+^ to NADPH for the synthesis of organic compounds. Arguably therefore, there is less need for plants to generate NADPH via NTH activity. In support of this hypothesis, transhydrogenase activity in the cyanobacterium *Synechocystis* is dispensable for its growth under autotrophic (normal light) conditions, while knockout of its transhydrogenase (*pntA*) gene leads to growth defects under low light conditions (Kamarainen et al., 2017). Thus, autotrophic organisms (including chemotrophs) may be less reliant on NTH than their heterotrophic counterparts. It should be noted that the presence of αβ- and βα-type *nth* genes in some species of primary algae (Table 1, Table S1) indicates that the ancestral plant cell possessed both types. Accordingly, these genes could have ended up in organisms such as *Plasmodium* (derived from secondary endosymbiosis) by horizontal gene transfer from the primary algal endosymbiont, perhaps reflecting the residence of *Plasmodium* NTH in the apicoplast (Saeed et al., 2020).

For this study we used a new algorithm for transmembrane helix (TM) prediction, DeepTMHMM (Hallgren et al., 2022). This software consistently predicted, across all eukaryotic taxa, a single stretch of 14 TM helices in αβ-type NTH proteins, or two stretches of typically 9 and 4 TM helices, respectively, in βα-type NTH proteins (Fig. 1B). This differed from TMHMM predictions (Krogh et al., 2001) that were more variable (e.g. see *Plasmodium* NTH (Saeed et al., 2020)) and that often calculated an odd number of TM helices in αβ-type NTH proteins (data not shown). The latter cannot be correct as it would position functional domains I and III on opposite sides of the membrane (Fig. 1A), rendering the enzyme non-functional. Thus, DeepTMHMM appears a more reliable TM prediction tool than TMHMM.

## Supplementary Material

Supplementary sequences

Table S1

Table S2

Table S3

## Figures and Tables

**Table 1:** Distribution of NTH isoforms across eukaryotes

	Lineage^1^			NTH isoform	
				αβ	βα
	
Amorphea	Opisthokonta	Metazoa		√	
		Fungi		√	
		Choanaflagellata		√	
		Ichtosporea			
	Amoebozoa			√	√
Archaeplastida	Chloroplastida	Chlorophyta		√	√
		Streptophyta			
	Rhodophyta			√	
	Glaucophyta				
Cryptista	Cryptophyceae			√	
Haptista	Haptophyta			√	
	Centrohelida				
TSAR	Telonemia				
	SAR	Stramenopila	Bigyra	√	√
			Gyrista	√	√
		Alveolata	Colpodellida	√	√
			Colponemida		
			Apicomplexa	√	√
			Cilliophora	√	√
			Perkinsidae		√
			Dinoflagellata	√	√
		Rhizaria	Cercozoa		
			Endomyxa	√	
			Retaria	√	
Excavates	Discoba			√	√
	Metamonada				√
	Malawimonadida				
CRuMs					

1 Classification according to (Adl et al., 2019; Burki et al., 2020).

**Table 2:** Distinct scenarios of *nth* gene loss/retention in apicomplexan and chromerid lineages

Lineage	αβ	NTH isoform/paralogue βα/I	βα/II
Chromerida	√	√	√
Gregarinia	√		√
Marosporida	√		√
Cryptosporidia		√	
Nephromycida			√
*Sarcocystidae*		√	√
*Eimeriidae*		√	
Haemosporida		√	
Piroplasmida			

## Data Availability

Generated Statement: The original contributions presented in the study are included in the article/supplementary material, further inquiries can be directed to the corresponding author/s.
