# Supplementary material for: NAD(P) transhydrogenase isoform distribution provides insight into apicomplexan evolution
Source: Front Ecol Evol. Author manuscript; Available in PMC 2023 Aug 14. (PMC7614955; doi:10.3389/fevo.2023.1216385)
Supplement: Supplementary sequences [file EMS183747-supplement-Supplementary_sequences.PDF]

$\beta\alpha$-type NTH sequences from Table S2

Siedleckia
MSLTMTGDFGLEEQYSNLELWVEMSYIGAAIFFTFSLRGLRHPESAKMGNIHGIIGMLLAVFATYASRFVSGQAMWIFFVAAGPPAILAALVAGRLRMTSLPEAVG
ALNACGGLSAALQAWAAYVSPYDKLKMEYMSPGSKFQVYQQITYVVSLVVGIITFTGSIIACLKLRGTIAGKPRTIPHRIYLSSFLLLCTVAIATVAAVFGLGHWTVGL
PCLAVCTFLSAVYGVLMVLAIGGADMPVAISILNSGSGWAGVFAGLAARSSVMVLAGAFVGASGIILSRIMCLSMNRSLLSVLLGGFGDKGAGHGHAGAAAAGA
GGADRGEATPASPEEVAAMMRAAKSVIVVPGYGMAVSKAQASVATLYEVLQKTGVQVRFAVHPVAGRMPGHMNVLLAEAGVPYDSVYAMDEINDDFDATDV
VLVVGANDTVNPAAERDPASAIAGMPVLRVWRATRTVVCKRSLGSGYAGVDNPLFFHDNNRMLFGDAKVTIDKVLVGLAVGASEAAADRLHPRAAPPAEAAYY
EPPERSSSLSPVRAHKSLEVGPASDDDEEEAAAASPPLPPLRRTIAVFREDVAQGEKRVAITPRGVQLFRKLNFRVLVESGAGRACHFEDDDYRAAGAEVCSGSQR
DTAGRTVAAGAEAPPSQSPPTGPAQLGGSAGDQSAGPELQDTSPAAAAGAGAAGAPTGTPSAAAAAAAASSTAAAALEGADVVVRLQRPPKPAAALDRLTRPRE
QLLLVVGRVDRETHRTFIEHAAQVGVTLASLEYLPRISSAQSMDPLSSMAKLAGYRAMMEGFHRFAQLPSGDITAAGSFPPATVLVLGAGVAGLEAVSVAHRLGA
DVRGFDIRLECKEQVESVGGKFLEMRFGDGPADTTTEGGYAKPMSAEFLRKEMELFMAQAKVCNIIVCTAAIPGRPAPLLLRKEHLEVMQRGSVVVDMAAQSG
GNCELTRPGETHVFAGRVEVVGSTDLLAGAATQASEMLCNNLANFLKHLRIDNDDAKQLPEGGGGGGGAGGGGGGGGGVPVGEDVAAAAAASVPEQLSLAD
PIVGSVVLAHRGVVVKAAPTAAAAKVVAVVVASSPPTSPARGGGGGGAAELTQEGESQTAKCGCLRCAGKRKARAGLNKQTFSRKYKGVVSLADILIVLLLAAGVA
FFSLTCPDYFAALLFVFALGCWVGYLLVWGVVPALHSPLMSVSNAISGVVMVGALLGISSYAHFDFQAQCRPARLDPLLIAAPDGTPGPMWWCQQRGAATISVN
SVAIAAAAINVAGGFTVTQKMLTMFRSSRTASSSK

Cephaloidophora
MSTFGDDGYERIQDLLAEHERWLGIVYVLGALCFSIAIFNFRKPSTSQMGNIFAIVGMATSLAGTLASTFVNGYGYWIFTGCFLSVGGIGIFMAVKVKMTNMPVFV
GFLNAMGGLAAALESLALYVSPIDEEQQNMEWELDWGDGGKKFQIYYTIFYLLGMIIGTITFSGSIVACLKLQGKFKFTTPRGKKINRILLKGRSLFTFILVAAFIATGF
VAGFWGLGVDPVGLTTFAVSMFLSGLYGIYFVLCIGGADMPVVISLLNSLSGWSTMFAGLAFGNELMIIAGSFVGASGLILSVLMCQAMNRSLKNVLVGGFGGDS
AVEEDRAKLDAVVVDVETVAKYICSANLVYVVPGYGMAVSQAQKQVAELGEIINASGRECRFAVHPVAGRLPGHMNVLLAEANVPYNYVDDITANGKLDETDVV
LVVGANDIYNPAAKFDKGSNIYGMEVLEVWNAKRTVVVKRSMRTGYAGIDNALFYYPQNSMLLGDAKEVIRSLADAVKHELAHGSEDARDAYMSGDCQRIAVR
VEDEKRLALAHANAVDDEDDEDDGPLHPSPMPVTVGFLREPKELNDRRIPLTPKDAIELMSKPWGIKKVLLQKGGDNDAMFADAGVLVCSRNEILTQSDIILSMTI
PNHADLDMVASHKMGGSSEIQDRAVFANLMVDGREVATEYRTRNIKPVVISVFGNPSGTSEAQEHARNLGISVLSMDIMPRVTTAQCMDVLSSSAKIAGFRAVI
EACNHYGRLIGAEITAAGSYRPAKCLVVGCGVAGLQAVGDAHRLGAEVRAFDVRKECADQVKSMGGKFLLMDFGDEDGAGAGGYANIMSPEFIEKEMALLHEQ
AKECDLYILTASIPGRKAPILLKSYHVASMKPGSVVVDLAAPSGGNCESTRPGENYTTDNGVIMIGCVDFVKTVSAPISSDMLSANLMNWFDYNVKSAGTLNVVD
VRDKIIRAIVLTHQGSKLWPAPRPPAPAPAVVIEDESEVDVVEVKQSWLSRRNTKFFNSSPADWIVLLFFFGFFALLGWSAPVSLNSKLMILMLSCWTGYLLVRGVT
VALHTPLMSLTNAISGVVIVAAMLSVSPLLAAGTSYSDNLCDDPSEANMFYCEERSIATVIIDSIALAAASINVVGGFAVTQRMLQMFKSK

Rhytidocystis 1
MVLPSFAMASSIDEESSSSCSTPFCPSIISPPPLRQLGINPEDAENILESDVWLQCIYVAASLFFILALRGLSHQETSKVGNLYGIVGMVAAIIATIVSPFVFDYGIWIFFV
VAVPPAVLAAGVSLYIRMTSMPQMVGILNACGGLAATLESFSLFFSPYEVGKQDYMYSDSAPRRYLQFQVYQTVFYLIGAVVGMVTFTGSLVACGKLSGRISSKPRI
MPGRVIYQPLLIMGILAMGALAGVYGFDDLPLGLVGLVVMTFLAAVYGVVFVMAIGGADMPVVISVLNSGSGWAGVFAGLTLQNSLMIIAGAFVGASGIILSYV
MCKAMNRSLYNVMIGGFGDSGPVASTKTYEGEASLTSPEEVAEWLVASESVIIVPGYGMAVSRAQHAVADMARVLVEMGVNVRFGVHPVAGRLPGHMNVLLA
EADVPYDIVLSMDEINGDFPTTDVVVVVGANDTVNPASQTEPGCAIWGMPVLEVWRAKHTVVLKRSLNVGYAGVDNPLFLYENNSMLLGDARKSLMAVVDGL
KDNNRKSGKLPMGKDSSNLRGGGIAEDSNLNVSTVVTENAVVIAMEDDEPAADPPTFHLGVLKETTWESSSGGAESIGSCADIGERRVSMSPKIAQKLKTSLGIG
VMLEAGAGTGSGFSDEVYLAAGCQIVPRKRILAECHVVAKVTAPTAEEVAEATLKDQVMVCGFLSPGSCPALLEQAAASGVTLMALDILPRLSTAQKMDVLSSTAK
LAGFRAVMEGFYHYGRLIGSEITAAGKYAPAKVLIIGAGVAGLQAVGDAHRMGADVRSFDIRLECKEQVESMGGKYLVMDFGEESGGGDGGYAKPMSEEFIKKE
MELFSQQAIECNIIITTAAIPGRPAPKLLKKEHVDLMQAGSVVVDLAAASGGNCDITRPGEVYVHNEKVTVIGLTDLASRMAPQATEMFANNIYHLLEHCGGSTKF
HVDMNDEIVRTITVARDFKVTYPPPKVTHPAPGQGIAKRSSLKYDDGAQKAANREAAAKPVGWMFRRYRGILSVMDIVVALIIAAFTALFATTAPPSLPPLLFVFM
LGCWVGYLLIWNVAPALHTPLMSVSNAISGVVLVGGMLGISPFAYDDLDDLPACSPDNTGAIPFYCRGRGVASVVLNAVAIAVASMNVFGGFTVTHRMLSMFKK
SSPSSASKNKVSRK

Rhytidocystis 2
MESSPFSSPRRLAIDAEDAENILEADVWMQCIYVACSLFFILALRGLGHQETSKVGNLYGVVGMVAALIATIISPFVFDYGIWIFFIVAVPPAVLAAGVSLYIRMTSMP
QMVGVLNACGGLAATLESFALFFSPYEVHKQDFMYSPSAPSRYTRFQVYQTVFYLIGAVVGMITFTGSLVACGKLSGRISSKPRIPPGRCVYQPLIVLGILAMGALA
GVYGFDDLPLGLVGLVVMTVLSAIYGVIFVMAIGGADMPVVISILNSGSGWAGVFAGLTLQNTLMIIAGAFVGASGIILSYVMCKAMNRSLYNVLVGGFGDSGHS
GSSKRYEGEANLTTPEEVAEWLIGAKSVIIVPGYGMAVSRAQHAVAEMAQLLLDRGVNVRFGVHPVAGRLPGHMNVLLAEADVPYDVVLSMDEINADFATTDV
AIIVGANDTVNPAAQTEPGCAIWGMPVLEVWRAQHTVVLKRSLNVGYAGVDNPLFLYENNSMLLGDARKSLTALVDGVKDTNTKASKLPTSKTSAGGGAEDSNL
NVSTVITENAVAISMDQPESPADPPRFHLGVLKEATSHNGATTCGTKEPNLSSILDVEERRVSMSPKIAQKLTSCMGVGVMVEFGAGRGSGFADESYVMAGCQM
TSRSKILKECRIVAKVTAPTTDDLLQCGTAVRDQVIVSGFLSPGSCKPLIDLAVSRGVTLMALDILPRISTAQKMDVLSSTAKLAGFRAVMEGFYHYGRLVGSEITAAG
KYPPAKILIIGAGVAGLQAVGDAHRMGADVRSFDIRLECKEQVESMGGKYLVMDFGDEADGGGGGGGYAKPMSDQFIQKEMELFSQQAVECNIIITTAAIPGRP
APKLLKKEHVDLMQPGSVVVDLAAASGGNCEVTRPGLVYVHNDKVTVVGLTDLPSRMAPQASEMFANNIYHLVEHCGGSSNFRVDMNDDIVRTITVARDFKLT
YPPAPIAHPVPPAAGKRASLRHDDLQAQHAAQLAGVKTTAWFDRRYRGVVSVRDFMLGLIIAAFTALFATTAPPSFPPLLFVFVLGCWVG

Rhytidocystis 3
MVASAEALLPSLSSTSPSCSPPFCWFTSARRLSVNPEDAENIVEADVWLQCVYVASSLFFILALRGLSNQESSKVGNIYGVVGMVAAILATLVSPFVFGYGIWVFFVV
AVPPAVLAAGVSLYIQMTSMPQMVGMLNACGGLAATLESFALFFSPYEVHKQDFMYGQGSPTRYTQFQVYQTVFYLIGGVVGMITFTGSIVACGKLSGHISSKPR
ILPGRAFYQPLLIAAIVGMGALAGVYGFNNFPLGLIGLSVMTVLAAVYGVLFVMAIGGADMPVVISVLNSGSGWAGVFAGLTLQNTLMIIAGAFVGASGIILSYVM
CKAMNRSLYNVMIGGFGDSGLTGSPKAYQGEANLTSSEEVSEWLVAAKGVIIVPGYGMAVSRAQHAVADLARLLIGRGVNVRFGVHPVAGRLPGHMNVLLAEA
DVPYDIVLSMDEINGDFPTTDVVIVVGANDTVNPAAQTEPGCAIFGMPVLEVWRAQRTVVLKRSLNVGYAGVDNPLFLYDNNAMLLGDARKSLSALVDGVRISN
TKAVAAPQDKRACRGGSGEESNLNVSTVVTENAVVIAMDGTEVPPQQLIFHLGVLREAPSLVDAPSCSPNIGCTMDIDERRASMSPSIAKKLSESLGIGVMVEPG
AGMGSGFSDEAYIHAGCQVVSRNVILQECQVVAKVTAPTIEEISQVALVDGQAFVSGFLSPGSCKPFLEKAASLGVTVMALDLLPRISTAQKMDVLSSTAKLAGFRA
VMEGFYHYGRLVGGEITAAGKYPPAKVLIIGAGVAGLQAVGDAHRMGADVRSFDIRLECKEQVESMGGKYLVMDFGNESGGGEGGYAKPMSEEFIKKEMELFS
QQASECNIIITTAAIPGRPAPKLLKKEHVDLMQAGSVVVDLAAASGGNCEVTRPGEVYVHNDKVTVVGLTDLASRMAPQATEMFANNIYHLLEHCGGSNGFHID
MTDVIVRAITVCHESKVTYPAPSPLLPPPAVSKRGSLKHNEQANQAAKQAANKPTAFVLRRYHGVVSIMDVVLALVIVAFTALFATTSPPSFPPLLFVFMLGCWVGY
LLIWNVAPALHTPLMSVSNAISGVVLIGGMLGISPYAHEDLEKLPACSATSSAAVPFYCRDRGVASVALNAVAIAVASMNVFGGFTVTHRMLSMFKKSSPSSKSTAK
G

Nephromyces 1

MDNWNDLINTLQAAVQLPVFVEVIYVASSLFFTLSLRGLSHPETSKMGNLFGIIGMLGAIIATLASDLVQDLEALIIAVIFLVILIISTIISFKIKMTSMPQLVGLLNAFGG
IAATLEALATFLKNDYPFSGNDYIFQMIFLEIGAAIGILTFAGSLVACGKLSGIIKGKPFIMPNRSIISSVIILAIIILATLSAVLHATNSAVAIILILLMIALSGIYGVLAVLAIGG
ADMPVVISILNSGSGWSGVFTGLMLNNTLMTIAGSFVGASGIILSYLMCKAMNRSILNVLIGGFGDTGAAVEKFEGTANIVDDKQATNYLLEAKSVIITPGYGMAV
SRAQHAVRDLTNLLRERGCNVRFGIHPVAGRLPGHMNVLLAEANIPYDIVLSMDEINPDFPSTDVSIIIGANDTVNPAAQTVPGFALAGMPVLEVWKSSQTIVLKR
SMNVGYAGVDNPLFLNSNNAMLLGDAKKSVEALCNNVRNATPVKKSANTTIVEMKKTLDTQSSSKSKDLKEVKPGYFKLGIINEIANSENRVSMHPSIVKKLKSSL
EIDVMIEVNAGMGANISDEAFEDAGAKIASKKEIFEEALVIAKINSFTNEEISMVTRPLEQVLICGFMSPNSNDEKIQTHIKHCQDLKVTTISLDVLPRITIAQKMDVL
SSMALLAGSRSVTEALSLYGRNFSQQLTAAGKYPQAKVFVIGAGVAGLQAIGDAHRLGAEVRAFDIRLECKEQVESLGGKYLVMEFDESGSGEGGYAKPMSPEFIA
KEMELFEEQSAECDIFITTASIPGRPAPKLMKKEHVDMMKHGSVIIDLAAGSGGNVEITKPGELYLYNNKVLVAGYTDLASRMAPQASEMFANNLFNLLGHMGG
GKMFGLDMKDQIIRSIVVTQQGEKLYPPPPPPKPAVTNQPTSTTNSNKQAGSPSEKKKSNNFFNNMNTKYYNIFTGWDMLILISIIVVTTILAAFSPANFPLLLFIFM
LGCWVGYLLIWNVTPALHTPLMSVSNAISGVVLLGALYGINVLPYQTFSVMQALNYAAVIFASFNICGGFAVTQRMLLMFVRTDS

Nephromyces 2
MDYLAMIDSLRPLPVYLEIIYVASSLFFTLSLRGLGHQETSKTGNLFGIIGMLTAIIASLASDLVGGTEAVIISAIFVVILFISTIITFKIKMTSMPQLVGLLNSFGGLAATLET
LGVYVKSHNPLMGENDYVFQMIFLEIGAAIGILTFTGSLVACAKLSGYIKSKPYIPPNRAIISSSIILIIVILATLSAVFHLASYAAGLVMFIIMIVLSGGYGILAVMAIGGAD
MPVVISILNSGSGWSGVFTGLMLNNSLMTISGAFVGASGIILSHLMCKAMNRSIVNVLLGGFGDTGAAVEKFEGTANIVDDKQAANWLVEAKSVIITPGYGMAV
SRAQHAVRDLTNELRARGCTVRFGIHPVAGRLPGHMNVLLAEANIPYDIVLSMDEINPDFPNTDVAIVIGANDTVNPAAQTVPGFALAGMPVLEVWKAKQTIVL
KRSMNVGYAGVDNPLFLNENNAMLLGDAKKSVEALCNNVRNTASSMQKSHKAMADLSPSNQSQATKIEEWEDLSKPGYFSLGILNEISDSEKRCAMHPSIVKKL
KKVLEIDVMVESGAGMGANIPDTSFENAGATIVTKKEIFENAKIIAKVGSFTQQEISLVSTPSEQILISGFMGPNSQDSETQDLIQHLLNNKVTILSLDVLPRITIAQK
MDVLSSMASLAGTRSVTEALSLYGRSFSQQVTAAGKYPQAKVFIIGTGVAGLQAIGDAHRLGAEVRAFDIRLETKEQVESLGGKYLIMEFEESGSGEGGYAKPMSP
EFIAKEMELFEDQARECDIFITTASIPGRPAPKLLKKYHVDMMKPGSIIIDLAALSGGNVEVTKPGQLYHYENKVYIVGYTDLASRMAPQASEMFANNMYNLLSHM
GGGKKFQMDMDDQIIRSITITSDGQKLYPPPPPPKPTTPVAPTSSKAIAQVSAPSKKEPGLFDNMNKKYYNFFTMWDVIILAMIIIVTTIFAIFSPSRFTELLFIFMLSC
WVGYLLIWNVTPALHTPLMSVSNAISGVVFLGALYDINHFGEYQVFTATNALNYLAVIFASFNICGGFAVTQRMLQMFVRT

Nephromyces 3
MADSAAVFFPDTSPPVYLECVYIAAALFFIMSLKGLAHPETAKIGNLYGVMGMLGAIIATWASGVLYGWSVWVLAIVVGLPSLLIIGVVLRIPMTSMPQMVGLLNS
FGGLASALEAFGLYFSGYEQRQEDLIVLPPSKEQVFRTVFYLFGATIGVLTFTGSLVACGKLAGCIASRPRVPPFRWFWNLLVVLGIVVLAVTTGILGLGDSLTGVVCFV
VMSVLSGVYGVMVVMAIGGADMPVVISVLNTGSGWSGVFAGLMLSNSLVIIAGSFVGASGIILSYVMCRAMNRSLMNVLLGGFGEGGGVGGVLERFEGEVTV
TDGARVAELLWSSRSVVIVPGYGMAVSQAQFATSELTEVLRGLGVRVRFGVHPVAGRMPGHMNVLLAEAKVPYDLALTMEEINPELPQTDVVIVIGANDTVNPA
AQLDLPGFALAGMPVIEVWKARQSVVLKRSLNVGYAGVDNLLFVKPNNSMLLGDAKQSVMDLVGRLRGMDSVHRRVSSTEASPIDGETVSPVVLGSPLEEATPS
YFCLGVLNECSLLERRVSLCPSMVGRMVQTMHVAVWVEWGAGVGSGFSDAEYVAQGAVMVTREALLASCRVIVRIHPFTVDELALLKSPEAQVLICGFMNPGS
LEEGVRGVIQRAVELKTTLLSLDVLPRISVAQKMDVLSSMALLGGNRAVVEALYYYRRPVGQQITAAGKYPQAKVFVIGAGVAGLAAVGEAHRAGAEVRGFDIRLE
CKEQVESLGGKYLVMEFDEDTSGEGGYAKPMSSEFIQKEMALFKAQAKECDIFITTAAIPGKPAPKLLLKEHVDLMKRGSVIVDLAAASGGNCEVTRPGEVYLYEDK
VQVVGLTDFPSRMAPQASEMFSTNVYNLLMHMGGGEKFGIQLDDTVVRTILIAYKGEKMFPAPRPAVAAVAPAIKAALPAPKSVDAAISSRLLESVQYRYGGVIS
WMNIFLIALIFFVTGILAAYSPKEFPPLLFVFVLGCWVGYMLIWNVTPALHTPLMSVSNAISGSVIIGGMLALSSYPNLSISERCAENNYPYCVENGVATIVLNFVAVG
LAVMNVSGGFAVTQRMLAMFKKS
