# Supplementary material for: NAD(P) transhydrogenase isoform distribution provides insight into apicomplexan evolution
Source: Front Ecol Evol. Author manuscript; Available in PMC 2023 Aug 14. (PMC7614955; doi:10.3389/fevo.2023.1216385)
Supplement: Table S1 [file EMS183747-supplement-Table_S1.PDF]

Table S1. Distribution of NTH isoforms across eukaryotes

Lineage					Isoform	
Amorphea	Ophistokonta	Metazoa		All	√	
		Fungi		>200	√	
		Choanaflagellata		Monosiga	√	
				Salpingoeca	√	
	Amoebozoa			Entamoeba		√
				Mastigamoeba		√
				Acanthamoeba	√	
				Cavenderia	√	
				Acytostelium	√	
				Polyspondynium	√	
				Dictostelium	√	
				Tieghemostelium	√	
				Heterostelium	√	
Archaeplastida	Chloroplastida	Chlorophyta		Bathycoccus		√
				Trebouxia	√	
				Gonium	√	
				Volvox	√	
				Chloropicon	√	
				Raphidocelis	√	
				Chlorella	√	
				Micratinium	√	
				Chlamydomonas	√	
				Micromonas	√	
				Auxenochlorella	√	
				Acetabularia	√	
	Rhodophyta			Chondrus	√	
				Gracialariopsis	√	
				Porphyra	√	
Cryptista	Cryptophyceae			Guillarda	√	
Haptista	Haptophyta			Chrysochromulina	√	
				Emiliania	√	
TSAR	SAR	Stramenopila	Bigyra	Cafereria	√	
				Hondaea	√	
				Blastocystis		√
			Gyrista	Ectocarpus	√	
				Thalassiosira	√	
				Phaedodactylum	√	
				Nannochloropsis	√	
				Fragilariopsis	√	
				Pelagophyceae	√	
				Tribonema	√	
				Crypsophaeum	√	
				Mayamaea	√	
				Aureococcus	√	
				Pelagomonas	√	
				Thalassiosira	√	

				Fragilaria	√	
				Seminavis	√	√
				Chaetoceros	√	√
				Nitzschia	√	√
				Pseudo-Nitzschia	√	√
				Fistulifera	√	√
		Alveolata	Colpodellida	Vitrella	√	√
				Chromera	√	√
			Apicomplexa	Gregarina	√	
				Porospora	√	√
				Siedleckia	√	√
				Selenidium	√	√
				Polyrhabdina	√	
				Ancora	√	
				Cephaloidophora		√
				Eimeria		√
				Cyclospora		√
				Toxoplasma		√
				Hammondia		√
				Besnoita		√
				Neospora		√
				Cystoisospora		√
				Cryptosporidium		√
				Plasmodium		√
				Hepatocystis		√
				Haemoproteus		√
				Nephromyces		√
				Cardiosporidium		√
				Rhytidocystis	√	√
			Cilliophora	Paramecium	√	
				Ichthyophthirius	√	
				Pseudocohnilembus	√	
				Stentor		√
				Tetrahymena		√
				Blepharisma		√
			Perkinsidae	Perkinsus		√
			Dinoflagellata	Symbiodinium	√	√
				Amoebophrya	√	√
				Polarella	√	
		Rhizaria	Retaria	Reticulomyxa	√	
				Plasmodiophora	√	
Excavates	Discoba			Stygiella		√
				Diplonema	√	
	Metamonada			Monocercomonoides		√
				Anaeramoeba		√
				Paratrimastix		√
				Carpedimonas		√
