# Supplementary material for: NAD(P) transhydrogenase isoform distribution provides insight into apicomplexan evolution
Source: Front Ecol Evol. Author manuscript; Available in PMC 2023 Aug 14. (PMC7614955; doi:10.3389/fevo.2023.1216385)
Supplement: Table S2 [file EMS183747-supplement-Table_S2.PDF]

Table S2. Distribution of NTH isoforms across Apicomplexa

Lineage	Genus	ID (if available)	Length (aa)	Isoform
Haemosporida	Plasmodium	PF3D7_1453500	1176	βα
	Hepatocystis	HEP_00305200	1252	βα
	Haemoproteus	Htart_000012700	1169	βα
Piroplasmida	Babesia	absent		
	Theileria	absent		
Eimeriidae	Eimeria	EMWEY_00011310	1106	βα
	Cyclospora	LOC34621507	1105	βα
Sarcocystidae	Toxoplasma	TGME49_318650-t26	1117	βα
		TGME49_301210	1244	βα
	Hammondia	HHA_318650	1019 (truncated)	βα
		HHA_301210	1245	βα
	Besnoita	BESB_044650	940 (truncated)	βα
		BESB_015750	1281	βα
Cryptosporidia	Cryptosporidium	Cgd1_990	1147	βα
		Cgd8_2330	1143	βα
Gregarinia	Gregarina	GNI_165100	1056	αβ
	Porospora	KAH0478387	1150	βα
		KAH0478386	1114 (truncated)	βα
		KAH0473193	1044	αβ
		KAH0486192	919 (truncated)	αβ
		KAH0486191 + KAH04086190 (tandem genes)	582 + 330	αβ
	Siedleckia	1	1287	βα
		2	990	αβ
		3	1062	αβ
		4	1059	αβ
	Selenidium	1	1118	αβ
		2	946 (truncated)	αβ
		3	494 (truncated)	βα
	Polyrhabdina	1	1061	αβ
	Ancora	1	1068	αβ
		2	1069	αβ
	Cephaloidophora	1	1144	βα
Marosporida	Rhytidocystis	1	1172	βα
		2	1038 (truncated)	βα
		3	1160	βα
		4	1103	αβ
		5	1056	αβ
Nephromycida	Cardiosporidium	KAF8821419	1099	βα

	KAF8819676	1097	βα
Nephromyces	1	1061	βα
	2	1054	βα
	3	1080	βα
