# Supplementary material for: NAD(P) transhydrogenase isoform distribution provides insight into apicomplexan evolution
Source: Front Ecol Evol. Author manuscript; Available in PMC 2023 Aug 14. (PMC7614955; doi:10.3389/fevo.2023.1216385)
Supplement: Table S3 [file EMS183747-supplement-Table_S3.PDF]

Table S3. TSAR-specific (excluding Apicomplexa) $\beta\alpha$-type NTH sequences

Lineage	Genus	ID	Length (aa)
Chromerida	Chromera	Cvel_6129	1205
		Cvel_12186	1090
	Vitrella	Vbra_8937	1109
		Vbra_16747	481 (truncated)
Stramenopila	Seminavis	CAB9501098	1097
	Fistulifera	GAX19811	1078
	Caetoceros	GFH58147	1079
	Nitzschia	KAG7349227	1085
Ciliophora	Blepharisma	CAG9328299	1056
		CAG9314130	1045
		CAG9335344	1032
	Stentor	OMJ82782	1055
		OMJ66621	1045
		OMJ73730	1032
Dinoflagellata	Amoebophrya	CAD7924036	1252
		CAD7954997	1377
		CAD7951945	1114
		CAD7967521	1138
	Symbiodinium	CAE7807561	1089
		CAE7330621	1255
Perkinsidae	Perkinsus	PMAR016593	1045
		PMAR016592	1050
		PMAR005427	1182
		PMAR010240	1069
